# Birefringent Bragg Grating in C-Shaped Optical Fiber as a Temperature-Insensitive Refractometer †

**DOI:** 10.3390/s18103285

**Published:** 2018-09-29

**Authors:** Rex Xiao Tan, Daryl Ho, Chun Ho Tse, Yung Chuen Tan, Seong Woo Yoo, Swee Chuan Tjin, Morten Ibsen

**Affiliations:** 1 School of Electrical and Electronics Engineering, Nanyang Technological University, 50 Nanyang Avenue, Singapore 639798, Singapore; darylho@ntu.edu.sg (D.H.); chtse@ntu.edu.sg (C.H.T.); seon.yoo@ntu.edu.sg (S.W.Y.); esctjin@ntu.edu.sg (S.C.T.); 2 Temasek Laboratories@NTU, 50 Nanyang Avenue, Research Techno Plaza, Singapore 637553, Singapore; yungchuen@ntu.edu.sg; 3 Optoelectronics Research Centre, University of Southampton, Southampton SO17 1BJ, UK; mi@orc.soton.ac.uk

**Keywords:** fiber sensors, fiber Bragg gratings, specialty fibers, refractometers

## Abstract

We demonstrate a simple-to-fabricate refractometer based on the inscription of fiber Bragg gratings in a special C-shaped optical fiber. The C-shaped fiber was drawn into shape using a quarter cladding removed preform of a commercial standard single-mode fiber by simple machining. The sensor did not suffer from cross-sensitivity of the refractive index with ambient temperature fluctuations, commonly occurring with many optical fiber refractometers. A refractive index sensitivity of 1300 pm per refractive index unit (RIU) was achieved without employing any additional sensitization techniques such as tapering or etching.

## 1. Introduction

Optical fiber sensors for chemical sensing have received increasing attention in recent years. This is mainly due to the maturity of optical fiber technologies and various inherent advantages of optical fiber devices over their electronics counterparts. Silica glass fiber sensors are immune to electromagnetic interference and are relatively inert to a wide range of chemicals. Naturally, surface-functionalized optical fiber refractometers have become the basis of many targeted chemical sensing applications [1,2,3]. Despite so, there remains the temperature cross-sensitivity problem that plagues many optical fiber-sensing schemes; for example, tapered and etched microfiber interferometric refractometers change in physical length with changes in temperature. This change in physical length results in a change in sensor spectral response, causing disruption of the capability of these sensors relying on spectral shifts for refractive index (RI) measurement. To overcome shortcomings such as this, researchers developed compensation methods [4,5,6] and designed temperature-insensitive sensor schemes [7,8], including birefringent fiber Bragg grating (FBG) refractometers.

Birefringent FBG sensors use fibers that are designed to expose orthogonal polarization modes differently to test substances, causing polarization mode drift measurable from the polarized Bragg reflections. This drift is not significantly affected by physical effects from temperature fluctuation, other than the possibility of actual RI change of test substances due to heat. Reported birefringent FBG sensor schemes include etched D-Shape [9], panda [10], and side-hole [11] FBGs that introduce polarized sensitivity by exposing guided orthogonal modes to the environment differently, through the etching of fibers into asymmetrical shapes. In addition, a rectangular [12] microfiber of a few micrometers that enabled a guided mode interaction with the environment without the need of etching was demonstrated. A side-hole fiber sensor scheme [13] was also implemented by pumping test substances directly into side holes close to the core of an FBG-inscribed side-hole fiber.

While showing promises of a temperature-insensitive measurement of RI, these sensors face several other challenges. Etched optical fibers sensors are difficult to fabricate and require precise control in order to replicate. Although methods of controlling etching [14] were studied, these processes add to the complexity of fabrication. Microfibers and most etched fiber sensors tend to be weak structurally and hence prone to breakages, rendering them unsuitable for a wide range of applications in environmental sensing. The side-hole fiber sensor was not physically thinned but does present challenges in environmental sensing applications without the assistance of a pump system. Lastly, polarization discrimination is not optimal for many of these designs, because of the fact that while one polarization mode is purposefully exposed, the orthogonal mode is also unintentionally exposed, as a result of the geometrical structure of the fibers after post-processing.

We propose a birefringent FBG sensor with the FBG directly written into a drawn-to-shape C-shaped optical fiber (C-fiber). This fiber allows consistent fabrication of the sensor with good repeatability, without the need for etching or tapering. The fiber is inherently sensitive to its environment off the drawing tower, much like drawn-to-shape microfibers, but with higher structural robustness. Because of its single-point exposure characteristic whereby only a quadrant of the cladding is modified, the sensor has good polarization mode discrimination-improving resolvability. It can also be easily mounted onto rigid surfaces using common adhesive on its unmodified side. This further enhances robustness and is suitable to be deployed for environmental sensing. As draw-tower grating [15,16] inscription research matures, it is conceivable that such a fiber sensor can be fabricated entirely, along with the fiber-drawing process.

## 2. Theory

### 2.1. FBG Reflections in Birefringent Waveguides

The back reflected wavelength, or Bragg wavelength ($\lambda_{Bragg}$), of a uniform FBG is dependent on the effective RI of the optical fiber waveguide. For unpolarized light interacting with a uniform FBG written in an unpolarized optical fiber, $\lambda_{Bragg}$ is a function of FBG fringe pitch (Λ) and effective RI ($n_{eff}$) of the optical fiber.

(1) $$\lambda_{Bragg}=2n_{eff}\Lambda$$

Unpolarized light reflected by a uniform FBG inscribed in an optical fiber of strong birefringence will result in two different Bragg wavelengths attributed to orthogonal polarization modes. For the sake of discussion, we name the orthogonal axes *x* and *y* and their corresponding Bragg wavelengths $\lambda_{Bragg_{x}}$ and $\lambda_{Bragg_{y}}$. These polarized FBGs can then be described by the following equation pair, modifications of Equation (1):

(2) $$\lambda_{Bragg_{x}}=2n_{eff_{x}}\Lambda \;;\;\lambda_{Bragg_{y}}=2n_{eff_{y}}\Lambda$$

where $\lambda_{Bragg_{x}}$ and $\lambda_{Bragg_{y}}$ are Bragg wavelengths along the *x* and *y* axes, $n_{eff_{x}}$ and $n_{eff_{y}}$ are effective RIs of the optical fiber seen by the guided modes along the *x* and *y* polarization axes, respectively. It is then apparent that the reflected light from the FBG now results in two spectrally separated polarized Bragg wavelengths. By altering the effective RI in both axes differently, the spectral separation between the polarized Bragg wavelengths changes accordingly.

### 2.2. Side-Exposed Single-Mode Fiber

The fundamental mode propagating in a single-mode fiber can be made loosely confined in the core, with a significant portion of the mode extended into the cladding, by the appropriate choice of the fiber parameters (i.e., core doping, core diameter, and operating wavelength). Ideally, removing the cladding of an optical fiber asymmetrically can expose only one polarization axis of the fundamental mode to the ambient environment. This results in one polarization mode becoming sensitive to its immediate ambient environment. However, in practice, both polarization modes will be exposed to the environment but to a different extent.

**Figure 1:**
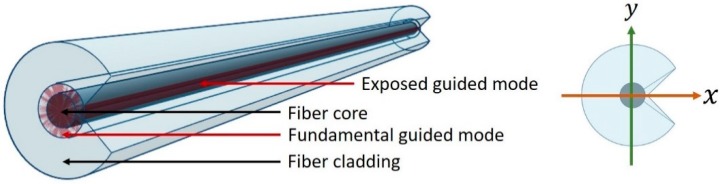
Schematic diagram of a C-shaped birefringent optical fiber.

The most common example of this class of fibers is the D-shaped fiber, often used in interferometric sensors [17] because of its fiber structure, partially exposing guided light to the environment. These fibers are usually polished or etched into a D-shape towards the core in order to expose the guided modes partially. The further the fiber cladding is removed towards the core, the more sensitive the fiber is to the ambient environment. A C-fiber, as depicted in Figure 1, operates on similar principles as the D-shaped optical fiber. However, the drawn-to-shape C-fiber results in guided light mode exposure without modifications, and the degree of exposure is much larger for one polarization than for the orthogonal polarization.

Assuming a Gaussian intensity-shaped beam propagating through the optical fiber, the polarization along the *x* axis will see one tail of the Gaussian beam exposed to and propagate in the fiber’s immediate environment. This exposed tail contributes to effective RI of its propagation in the waveguide. The extent of exposure determines the degree of this effect, which implies a change in birefringence of the waveguide.

### 2.3. Fiber Bragg Grating in a C-Fiber

**Figure 2:**
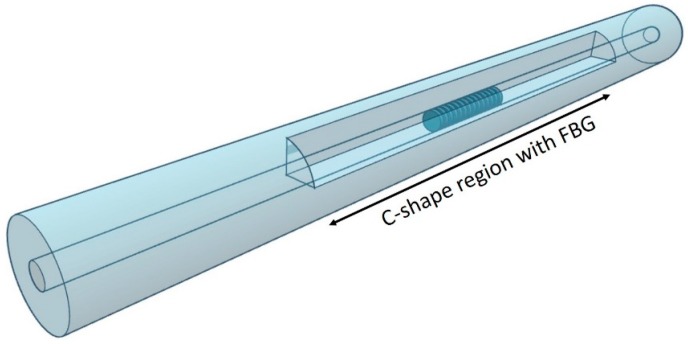
Schematics of a C-fiber with fiber Bragg grating spliced to standard single-mode fibers as an inline refractive index sensor.

Because of the environment-dependent birefringent properties of the C-fiber, FBGs inscribed in the fiber will naturally be both birefringent and sensitive to ambient RI. As such, the spectral characteristics of reflected light from the FBG exhibits twin reflection peaks, corresponding to polarizations along the *x* and *y* axes, respectively. A sensor can then be formed by splicing the C-shaped FBG to standard single-mode fibers (Figure 2) and connecting it to an interrogation circuit.

### 2.4. Temperature Fluctuation Insensitivity

The Bragg wavelength of a single FBG, regardless of polarization, is affected by temperature changes that the host optical fiber experiences. This effect is well studied and is the basis of FBG thermometer designs [18]. The governing relationship of the Bragg wavelength shift resulting from a temperature change is as follows:

(3) $$\frac{\Delta \lambda_{Bragg}}{\lambda_{Bragg}}=\left(\alpha_{\Lambda }+\alpha_{n}\right)\Delta T$$

where $\alpha_{\Lambda }$ is the optical fiber thermal expansion coefficient, and $\alpha_{n}$ is the thermo-optic coefficient. This effect often causes accuracy and cross-sensitivity issues for FBG-based refractometers that depend on a single $\lambda_{Bragg}$ acting as an indicator of RI change by its absolute wavelength shifts [19,20]. The Bragg wavelength shift from the effect of temperature fluctuation, in this case, is coupled with that of ambient RI, resulting in a significant measurement error. However, in our proposed scheme, the measurement of RI is inferred from orthogonal polarization difference, which is not affected by the temperature significantly, as both polarizations undergo almost identical shifts with changes in temperature but differing shifts with the change of RI.

## 3. Sensor Fabrication and Experiment

### 3.1. Sensor Fabrication

#### 3.1.1. C-Fiber Fabrication

**Figure 3:**
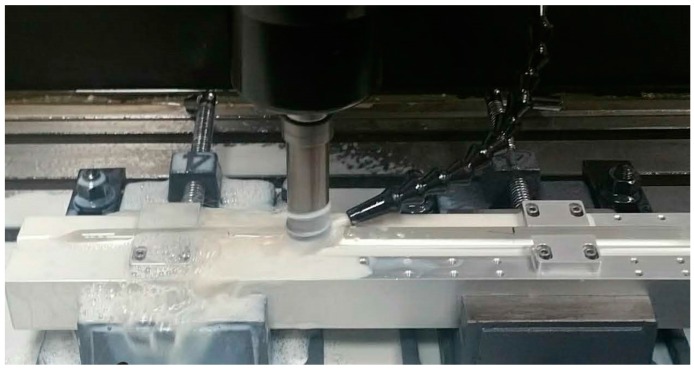
Milling of the preform on a customized milling stage with Standard Series 10 Rotary Knee mill from Sonic-Mill.

**Figure 4:**
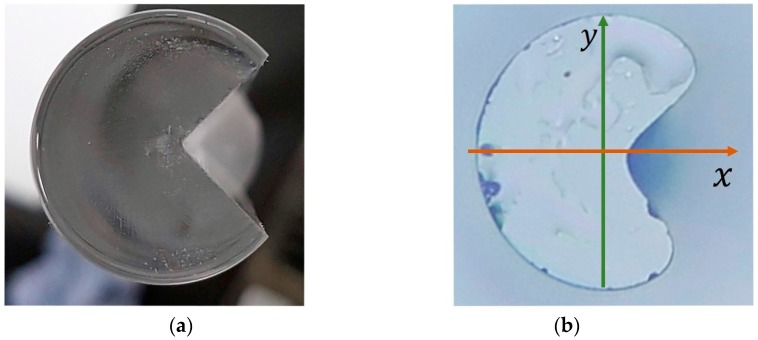
(**a**) Preform of standard single-mode fiber with a quadrant cut out. (**b**) Fiber-end image of the C-shaped fiber.

The C-fiber was fabricated in-house using a quarter cutout Germanium-doped single-mode fiber preform (G.652D) of 19.95 mm outer diameter, as shown in Figure 4a, where the preform core was exposed at the apex of the triangle. The cutout of the preform was an isosceles triangle with apex at 45° and equal sides of 9.35 mm. The preform was placed onto a customized jig which held it firmly in place, and the quarter was milled away from the workpiece with a high-precision knee mill (Sonic-Mill Rotary Series 10 Ultrasonic Machine). The milling bit used was of 25 mm diameter, operating at a spin speed of 2000 rpm and moving in repeated passes across the preform horizontally in a controlled manner at a speed of 30 mm/min. Each pass of the milling bit cut 1 mm of the preform, and, hence, multiple cuts were made to precisely remove the predetermined amount of material from the preform with accuracy of ±100 μm tolerance. During the milling process, coolant was applied by a jet spray between the milling bit and the workpiece (See Figure 3).

This process resulted in the apex of the triangle cutoff to be exactly touching the edge of the core (See Figure 4a). The milled preform was then loaded into a fiber drawing tower and drawn into a fiber of outer diameter of 122 μm, at a tower furnace temperature of 2000 °C, with a draw speed of 10 m/min. The entire fabrication process was done in-house with an SG Controls fiber drawing tower.

**Figure 5:**
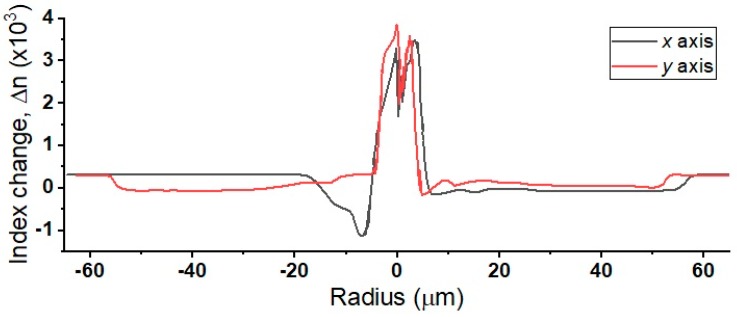
Index profile of a C-Shaped optical fiber along the *x* and *y* axes.

Because of fluid dynamics during the fiber drawing process, the exposed point became coated with a thin layer of mixed cladding/core material, resulting in extremely thin higher RI cladding where the fiber core was exposed. This thin layer of coating allowed the fiber to retain proper light-guiding characteristics, while also allowing guided modes to interact with the environment. Figure 5 shows the linear index profiles (measurement equipment: IFA-100) of both the *x* and *y* axes. The first index drop indicates the transition from the index oil to the cladding of the optical fiber. Index oil was required in the measurement equipment both as a medium that guides light towards the fiber, and to suspend the fiber in place for measurement. For the *x* axis, it is obvious that cladding was thinned to an effective thickness of less than 5 μm, resulting in guided modes extending out of the fiber into the ambient environment.

The fundamental mode is more exposed to the environment along the *x* axis than along the *y* axis. Therefore, the two orthogonally polarized components in the fiber behave differently, depending on the degree of exposure to the environment.

It is apparent that the performance and repeatability of the sensor is dependent on the physical structure of the optical fiber. Once the process of milling and fiber-drawing is optimized, the undesired variation of the fiber structure can be limited. The milling machine used in this work has an accuracy tolerance of ±100 μm, which translates to a less than ±1 μm variation at the fiber level, given that the preform was >100× larger than the fiber. The fiber-drawing process is a matured technology, where the fiber tower furnace temperature can be controlled at a resolution of 1 °C. Therefore, variation of the fiber’s physical structure is minimized. However, as the fiber sensor performance can still be affected by small differences, just like other fiber refractive index schemes, each fabricated sensor must be individually characterized.

#### 3.1.2. FBG Inscription

A 10 mm FBG of 536.45 nm fringe pitch was inscribed into the middle of an 80 mm segment of C-fiber. The grating inscription was carried out with a scanning phase mask setup, employing a 244 nm frequency-doubled Argon ion laser. The C-fiber was carefully orientated on the fiber stage for the UV laser beam to incident squarely on the round side of the fiber, ensuring that light was not diffracted by the steep corners of the C-fiber. Using deionized (DI) water (Measured RI: 1.333, Measurement equipment: DR201-95 (KRUSS GmbH, Hamburg, Germany)) as the reference ambient environment, the resulting grating had polarized Bragg wavelengths of 1550.435 nm in the *x* direction and 1553.212 nm in the *y* direction.

### 3.2. Experiment

**Figure 6:**
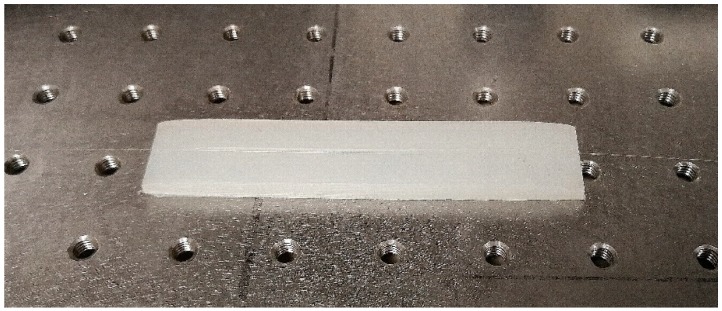
C-Shape optical fiber with FBG inscribed affixed to a nylon block with a V-groove.

The C-fiber FBG sensor was carefully orientated and affixed using common epoxy to a 3D printed nylon flexible holder which consisted of a V groove (see Figure 6) so that the thin cladding side of the fiber was unaffected and was facing outwards, exposed to the environment.

**Figure 7:**
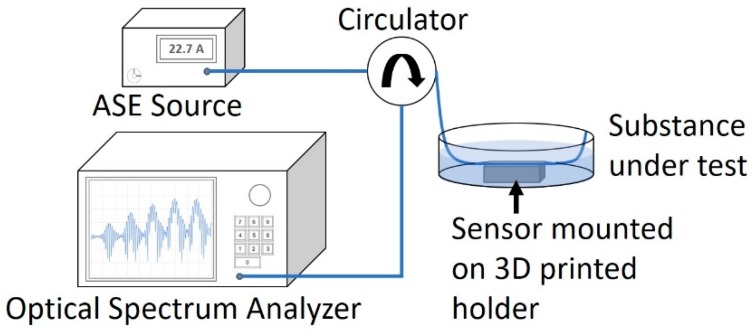
Experimental setup.

The fiber sensor was interrogated according to Figure 7. Measurements were taken for five substances, i.e., DI water of RI 1.333 and four glycerin solutions diluted to RIs of 1.340, 1.360, 1.380, and 1.410. The RI range chosen is much lower than the fiber core so as to prevent excessive out-coupling of light from the fiber. The light source used was an amplified spontaneous emission source, with significant optical power in the wavelength range of 1480 nm to 1620 nm.

The sensor was flushed with DI water after each measurement, and the experiment was repeated three times in a 25 °C environment. In consideration of temperature bias, each reading was taken after allowing the fiber to sit in the test substance for exactly 1 h. All three sets of measurement exhibited largely identical results, indistinguishable within the resolution of the optical spectrum analyzer (Model: ANDO AQ6317B; Wavelength resolution: 0.01 nm; Power resolution: 0.01 dB).

The sensor was then immersed in DI water at a temperature in the range of 25 °C to 34 °C, reached by heating with an electrical hot plate, which was then power-lowered for the water to cool very gradually, at approximately 0.2 °C/min, to the temperatures of interest. This chosen temperature range ensured that the result for this measurement was not significantly affected by the minor change in DI water’s RI (<10^−3^) due to temperature change. The temperature range was approximated [21] using Equation (4)

(4) $$n_{1}\left(\lambda ,t_{1}\right)-n_{2}\left(\lambda ,t_{2}\right)<10^{-3}$$

(5) $$n_{i}\left(\lambda ,t_{i}\right)=A\left(t_{i}\right)+\frac{B\left(t_{i}\right)}{\lambda^{2}}+\frac{C\left(t_{i}\right)}{\lambda^{4}}+\;\frac{D\left(t_{i}\right)}{\lambda^{6}}$$

where *λ* is the operating wavelength, $t_{i}$ is the temperature in °C, and A, B, C, D are temperature-dependent Cauchy coefficients determined experimentally in reference [16]. It was then found that the index change was 9.9 × 10^−4^ RIU for a temperature change of up to 9 °C, from 25 to 34 °C, and hence, measurements were taken at 25, 28, 31, and 34 °C and analyzed for the temperature effect on RI measurement.

## 4. Results and Discussions

### 4.1. Refractive Index Measurement

**Figure 8:**
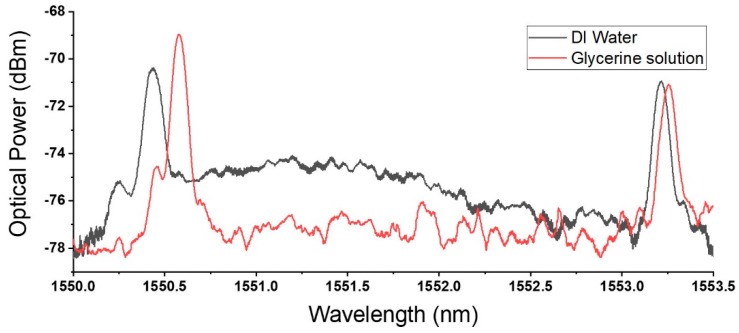
Spectral response in deionized water and a glycerin solution with refractive index of 1.410.

The spectral responses for deionized water and a glycerin solution with RI of 1.410, using the proposed sensor, are shown in Figure 8. It is obvious from the spectral plot that, while the FBG reflection spectrum for both orthogonal polarizations redshifted with the increase in ambient RI, *x* and *y* polarizations shifted with different magnitude because of differing extents of exposure to the ambient environment, forming the basis of RI measurement of this sensor.

It was observed from the spectrum (Figure 8) that there was Mach–Zehnder interference arising from the interference of light of differing phases as they coupled from the C-fiber into the standard single-mode fiber. Although this is the basis of some optical fiber refractometers [22], this effect was regarded as noise in this sensor. However, the visibility of the resulting Mach–Zehnder interference was small and did not affect Bragg reflection. Therefore, the effect of this interference could be ignored.

When submerged in DI water, the Bragg wavelengths of the *x* and *y* polarizations were 1550.435 nm and 1553.212 nm, respectively, with a difference between them of 2.777 nm. Because of the relationship between Bragg wavelength and effective RI of waveguide described in Equation (2), it was calculated that, with ambient RI of 1.333, $n_{eff_{x}}$ was 1.445, and $n_{eff_{y}}$ was 1.448. This was higher than the expected birefringence of an optical fiber with pure silica cladding and could be attributed to the induced stress in the drawn fiber due to the irregular shape of the preform.

As the DI water was drained and replaced with the glycerin solution with RI 1.410, the Bragg wavelengths of the *x* and *y* polarizations became 1550.578 nm and 1553.255 nm, respectively. The difference between the Bragg wavelengths of the orthogonal polarizations was reduced to 2.677 nm. This translated to an RI sensitivity of 1300 pm/RIU, within the range of RI from 1.333 to 1.410. The same measurements were acquired for a total of five different ambient RI, from 1.333 to 1.410.

**Figure 9:**
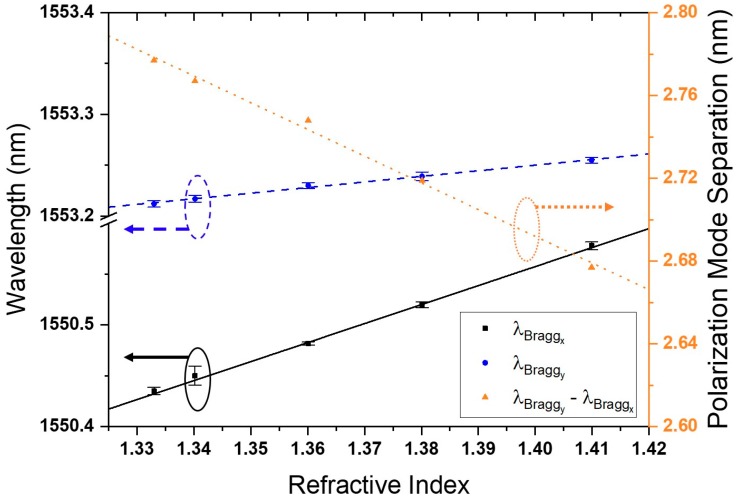
Sensor response to changes in ambient refractive index.

Figure 9 shows the linear redshift of the Bragg wavelength for both polarizations, along with increasing ambient RI. However, $\lambda_{Bragg_{x}}$ redshifted more with the increase of ambient RI than $\lambda_{Bragg_{y}}$. Therefore, the distance between the Bragg wavelength of both polarizations decreased linearly and was indicative of the ambient RI change.

### 4.2. Temperature Response

**Figure 10:**
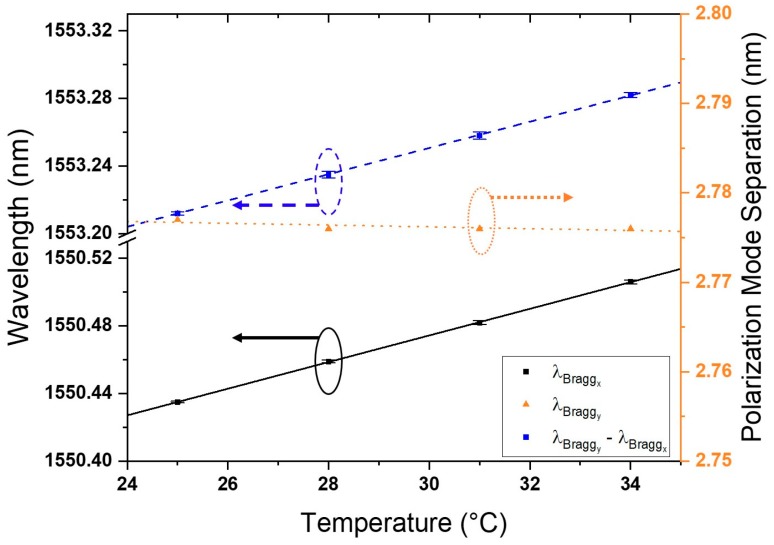
Sensor response to changes in ambient temperature.

The sensor assembly was placed in a water bath with temperatures varied from 25 to 34 °C, as determined in Section 3. As the sensor was bonded to the nylon holder, the temperature response was a contribution of the material properties of fiber, holder, and adhesive used. As temperature increased, the Bragg wavelengths of both polarizations redshifted. By considering the evolution of the spectral difference between the Bragg wavelengths of the orthogonal modes (Figure 10), it was apparent that a small ambient temperature fluctuation within 9 did not significantly influence RI measurement capability.

The slight increase in the Bragg wavelength separation can be attributed to the small increase in the RI of the DI water as it heated up. According to the approximation discussed in Section 3.2, the RI for DI water due to a 9-degrees increase of the temperature will show approximately an 8.0 × 10^−4^ RIU increase. A 1 pm change in polarization mode separation was measured over the 9 degrees of temperature difference. As previously established, the sensor had a sensitivity of 1300 pm/RIU. A 1 pm change in polarization mode separation translates to 7.69 × 10^−4^ RIU increase, close to the approximated increase of 9.90 × 10^−4^ RIU. Therefore, the slight decrease in mode separation could be attributed to the RI change of DI water with temperature. It is noteworthy that, if a larger change in temperature is considered, the RI of ambient liquid will be significantly altered and will result in a prominent change in the Bragg wavelength separation of the sensor.

## 5. Conclusions

A viable C-fiber birefringent FBG refractometer was presented. The FBG in the special optical fiber is naturally sensitive to the ambient environment because of the geometry of the C-fiber, without the need for post-processes such as tapering or etching. The sensor is easy to fabricate and retains the fiber’s physical robustness while achieving a sensitivity to ambient RI of 1300 pm/RIU. While this sensitivity is lower than that achieved by many other reported fiber sensor schemes, the C-fiber FBG refractometer is still a good candidate for many applications as an RI sensor for its ease of fabrication, potential for on-tower fabrication of distributed array, physical robustness, and ability to be decoupled from temperature fluctuation. Being only modified on and required to be exposed at one quadrant of the optical fiber, one can easily integrate the sensor into physical structures using simple adhesives, further increasing its physical robustness and suitability for environmental sensing.
